# Ultrarapid Inflammation of the Olfactory Bulb After Spinal Cord Injury: Protective Effects of the Granulocyte Colony-Stimulating Factor on Early Neurodegeneration in the Brain

**DOI:** 10.3389/fnagi.2021.701702

**Published:** 2021-06-25

**Authors:** Muh-Shi Lin, I-Hsiang Chiu, Chai-Ching Lin

**Affiliations:** ^1^Division of Neurosurgery, Department of Surgery, Kuang Tien General Hospital, Taichung, Taiwan; ^2^Department of Biotechnology and Animal Science, College of Bioresources, National Ilan University, Yilan, Taiwan; ^3^Department of Biotechnology, College of Medical and Health Care, Hung Kuang University, Taichung, Taiwan; ^4^Department of Health Business Administration, College of Medical and Health Care, Hung Kuang University, Taichung, Taiwan

**Keywords:** subjective cognitive decline, neurodegenerative disease, olfactory dysfunction, olfactory bulb, spinal cord injury, neuroinflammation, granulocyte colony stimulating factor

## Abstract

The correlation among olfactory dysfunction, spinal cord injury (SCI), subjective cognitive decline, and neurodegenerative dementia has been established. Impaired olfaction is considered a marker for neurodegeneration. Hence, there is a need to examine if SCI leads to olfactory dysfunction. In this study, the brain tissue of mice with spinal cord hemisection injury was subjected to microarray analysis. The mRNA expression levels of olfactory receptors in the brain began to decline at 8 h post-SCI. SCI promoted neuroinflammation, downregulated the expression of olfactory receptors, decreased the number of neural stem cells (NSCs), and inhibited the production of neurotrophic factors in the olfactory bulbs at 8 h post-SCI. In particular, the SCI group had upregulated mRNA and protein expression levels of glial fibrillary acidic protein (GFAP; a marker of astrocyte reactivation) and pro-inflammatory mediators [IL-1β, IL-6, and Nestin (marker of NSCs)] in the olfactory bulb compared to levels in the sham control group. The mRNA expression levels of olfactory receptors (*Olfr1494*, *Olfr1324*, *Olfr1241*, and *Olfr979*) and neurotrophic factors [brain-derived neurotrophic factor (BDNF), glial cell-derived neurotrophic factor (GDNF), and nerve growth factor (NGF)] were downregulated in the olfactory bulb of the SCI group mice at 8 h post-SCI. The administration of granulocyte colony-stimulating factor (G-CSF) mitigated these SCI-induced pathological changes in the olfactory bulb at 8 h post-SCI. These results indicate that the olfactory bulb is vulnerable to environmental damage even if the lesion is located at sites distant from the brain, such as the spinal cord. Additionally, SCI initiated pathological processes, including inflammatory response, and impaired neurogenesis, at an early stage. The findings of this study will provide a basis for future studies on pathological mechanisms of early neurodegenerative diseases involving the olfactory bulb and enable early clinical drug intervention.

## Introduction

**FIGURE 1:**
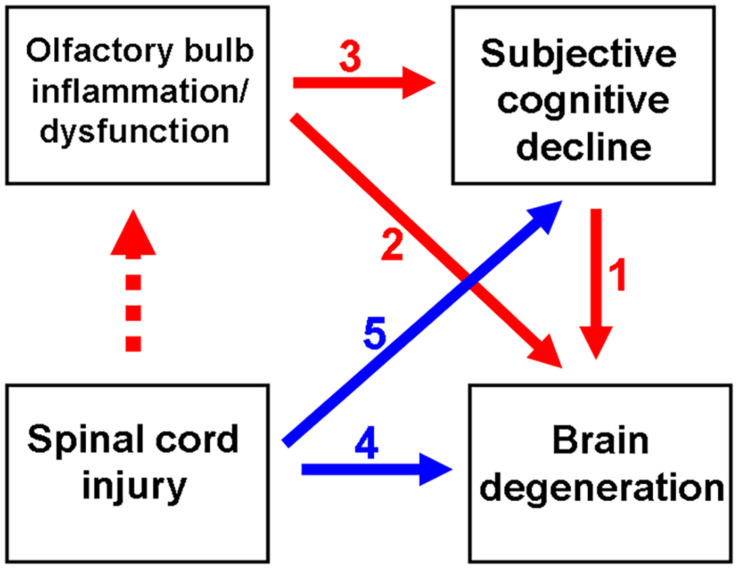
Schematic diagram depicting spinal cord injury (SCI)-mediated olfactory dysfunction and the subsequent subjective cognitive decline (SCD)/neurodegenerative dementia. Olfactory dysfunction is an early indicator of neurological diseases, including SCD and neurodegenerative dementia. Previous studies have reported the correlation between SCD and neurodegenerative disease (ND) (indicated as red arrow no. 1). Generally, olfactory impairment is involved in the progression of ND (indicated as red arrow no. 2) or the progression of SCD to ND (from red arrow no. 3 to 1). Thus, SCI promotes ND (indicated as blue arrow no. 4), as well as the progression from initial SCD to ND (from blue arrow no. 5 to red arrow no. 1). This study demonstrated that SCI promotes neuroinflammation in the olfactory bulb at an ultrarapid stage after SCI. Thus, SCI may mediate the pathological mechanisms of neurodegeneration.

The cognitive performance of patients with subjective cognitive decline (SCD) in the objective cognitive examination is within the standard range (Jessen et al., 2014). SCD is considered to be the preclinical stage of Alzheimer’s Disease (AD). Approximately 25% of patients with SCD may develop mild cognitive impairment (MCI). The risk of developing dementia within 5 years in patients with SCD was twofold higher than that in patients without SCD (Mitchell et al., 2014) (red arrow numbered 1, Figure 1). Therefore, there is a need to further examine the clinical characteristics of patients with SCD. Obscure cognitive symptoms at early stage can be alleviated (Si et al., 2020). Previous studies have reported that SCD is associated with increased glial activation and consequently increased inflammation in the brain (Nordengen et al., 2019; Si et al., 2020).

As shown in Figure 1 (red arrow numbered 2), the manifestation of olfactory dysfunction is reported in various neurological diseases, such as Parkinson’s Disease (PD) (Altinayar et al., 2014), AD (Yoo et al., 2018), stroke (Wehling et al., 2015), and major depression disorder (Negoias et al., 2016; Croy and Hummel, 2017). Additionally, olfactory dysfunction is highly correlated with SCD (Risacher et al., 2017; Jobin et al., 2021; Wang et al., 2021), which may further progress to MCI and neurodegenerative dementia (Devanand et al., 2015; Fullard et al., 2016; Roberts et al., 2016; Dintica et al., 2019; Yahiaoui-Doktor et al., 2019) (red arrow numbered 3 to 1, Figure 1). Olfactory deficits increase the risk of developing AD dementia from MCI by four to five times (Devanand et al., 2008). Thus, olfactory impairment is suggested to be a marker for the early detection of cognitive decline and AD dementia (Devanand et al., 2015; Fullard et al., 2016; Roberts et al., 2016).

Traumatic spinal cord injury (SCI) leads to neurological deficits or chronic disability. Acute SCIs involve both pathophysiological primary and secondary mechanisms of injuries. Primary injuries involve damages to the neural structures (such as the cell membranes, myelin, axons, and microvessels) and can contribute to the exacerbation of secondary injuries (Kwon et al., 2004). Secondary SCIs include neuroinflammation, production of free radicals, hyperoxidation, and neuronal apoptosis (Tator and Fehlings, 1991; Houle and Tessler, 2003), which lead to irreversible neurological deficits in patients with SCI.

In addition to the spine, SCIs can adversely affect the brain and consequently promote inflammation in the brain (Wu et al., 2014) (blue arrow numbered 4, Figure 1). The enhanced production of pro-inflammatory cytokines and neurotoxic molecules post-SCI promotes inflammation in the brain (Tian et al., 2007). In the rat models of SCI, microglial activation promotes chronic inflammation in the thalamus, hippocampus, and cerebral cortex (Wu et al., 2014). Furthermore, suppressed neuroprotective mechanisms may contribute to the exacerbation of cerebral damage post-SCI. The expression of brain-derived neurotrophic factor (BDNF) is downregulated for at least 1 week after SCI, which adversely affects the plasticity of the rat hippocampus (Fumagalli et al., 2009). SCIs are reported to result in cognitive impairment (Craig et al., 2017; Sachdeva et al., 2018; Nightingale et al., 2020) and contribute to the development of neurodegenerative diseases, such as AD (Yeh et al., 2018) and PD (Yeh et al., 2016) (blue arrow numbered 5 to red arrow numbered 1, Figure 1). Olfactory dysfunction can predict neurodegeneration. However, the predictive value of olfactory dysfunction for cognitive impairment and subsequent dementia in patients with SCI has not been previously reported.

Granulocyte colony-stimulating factor (G-CSF) or colony-stimulating factor 3 (CSF3) is widely used for the clinical treatment of patients with neutropenia after chemotherapy, radiotherapy, or hematopoietic stem cell transplantation (Welte et al., 1996). The functions of G-CSF are to activate hematopoietic stem cells and stimulate the proliferation, differentiation, and maturation of neutrophils in the bone marrow (Holig, 2013). G-CSF is reported to exert neuroprotective effects in neurodegenerative diseases, including PD and AD (Tsai et al., 2007; Prakash et al., 2013a, b; Safari et al., 2016). The plasma concentrations of G-CSF in patients with early-stage AD are lower than those in healthy individuals (Laske et al., 2009). The administration of G-CSF improves the cognitive functions in patients with early-stage AD (Sanchez-Ramos et al., 2012). Moreover, G-CSF is reported to alleviate depression and motor function in rat PD models and increase the density of neurons in the substantia nigra pars compacta (SNpc) (Prakash et al., 2013a). Additionally, G-CSF can restore the functions of striatum and SNpc in the 1-methyl-4-phenyl-1,2,3,6-tetrahydropyridine (MPTP)-induced PD mouse model (Song et al., 2011). Furthermore, G-CSF exhibits anti-inflammatory and neuroprotective effects in stroke and SCI (Kadota et al., 2012; Guo et al., 2015; Cui et al., 2016; Weise et al., 2017).

As shown in Figure 1 (dotted red arrow), the effect of SCI on olfactory function has not been elucidated. This study examined the effect of SCI on neuroinflammation and the levels of olfactory receptors, neurotrophic factors, and neural stem cells (NSCs) in the mouse olfactory bulb. The findings of this study suggested that SCI promotes olfactory dysfunction, which indicated a correlation between SCI and neurodegenerative diseases. This study also used a mouse spinal cord hemisection model to investigate the therapeutic effects of G-CSF on the SCI-induced pathological changes in the olfactory bulb.

## Materials and Methods

### Animals

Adult BLTW: CD1 (ICR) male mice aged 8 weeks and weighing 31–33 g were obtained from the BioLASCO Experimental Animal Center (Taiwan Co., Ltd., BioLASCO, Yilan, Taiwan). The animals were housed in cages (five animals per cage) under a regular circadian cycle with free access to food. This study was performed according to the guidelines outlined by the Experimental Animal Laboratory and approved by the Animal Care and Use Committee at National Ilan University, Yilan, Taiwan (IACUC Approval No.: 106-14).

### Experimental Grouping

The animal model of spinal cord hemisection was established as described previously (Lin et al., 2011). Briefly, the mice were divided into the following four groups: sham-operated control (sham control group), animals underwent laminectomy (*n* = 18); vehicle-treated SCI group (SCI group) (*n* = 18), animals underwent spinal cord hemisection and administered with physiological saline; SCI + G-CSF i.p. group, SCI mice intraperitoneally administered with G-CSF (*n* = 18); and SCI + G-CSF oral group, SCI mice orally administered with G-CSF (*n* = 15). Microarray, mRNA, protein, and immunofluorescence analyses were performed using three, six, six, and three mice from the sham control, SCI, and SCI + G-CSF i.p. groups, respectively. As the SCI + G-CSF oral group comprised three mice less than those in the other groups, microarray analysis was not performed for this group.

### Spinal Cord Hemisection

The animals were anesthetized using isoflurane, placed in a stereotactic apparatus (David Kopf Instruments, Tujunga, CA, United States) to secure the spinal cord, and subjected to posterior decompression. Laminectomy was performed at the 9th to 10th thoracic vertebrae with undisturbed intact dura under a dissecting microscope. For spinal cord hemisection, the guide of the wire knife was positioned along the vertical plane close to the lateral surface in the lower thoracic level of the spinal cord. The knife was turned medially and extended 1.5 mm. Next, the guide was lifted 4.0 mm to hemitransect the spinal cord. The sham group only underwent laminectomy but not hemisection. The wound was closed in layers using sutures. The animals were allowed to recover on a heating pad at 36.5°C and fast for 3 h post-surgery. For postoperative care, the animals were subcutaneously injected with saline for rehydration. The mice were returned to their preoperative housing conditions after surgery. The whole brain and the olfactory bulb were excised from mice in all four groups for microarray and gene expression analyses at 8 h post-hemisection.

### Administration of G-CSF in SCI Mice

The SCI + G-CSF i.p. and SCI + G-CSF oral groups were intraperitoneally and orally administered with G-CSF (50 μg/kg bodyweight) at 30 min post-SCI. Meanwhile, the SCI group was intraperitoneally administered with physiological saline at 30 min post-SCI. The mice in the sham control group were not administered with physiological saline or G-CSF.

### Preparation of Recombinant G-CSF

Recombinant G-CSF (rG-CSF) was synthesized in our laboratory. Briefly, U-87 MG cells (Bioresource Collection and Research Center, Hsinchu, Taiwan) were cultured in Eagle’s Minimum Essential Medium (11700-077) (Gibco^TM^, Thermo Scientific, Waltham, MA, United States) supplemented with 10% fetal bovine serum (A15-101) (PAA Cell Culture Company, BioPath Stores, Cambridge, United Kingdom). Total RNA was extracted from the cells (1 × 10^6^–1 × 10^7^ cells) using TRI Reagent^®^ RNA isolation reagent (15596-018) (Invitrogen, Carlsbad, CA, United States), following the manufacturer’s instructions. The RNA was reverse-transcribed to cDNA before PCR. The PCR product was purified from the agarose gel and subcloned into the T&A vector (Yeastern Biotech, Taipei, Taiwan). The recombinant vector was transformed into competent *Escherichia coli* RR1 cells. The final sequences with a size of 525 bp were purified from an agarose gel and subcloned into the pET-24a(+) expression vector (Novagen, Merck KGaA, Darmstadt, Germany) to obtain the pET-24a(+)-rG-CSF construct. Next, the pET-24a(+)-rG-CSF plasmid was transformed into *E. coli* BL21 Codon Plus^®^ (DE3)-RIPL cells. rG-CSF was purified using affinity chromatography using the 6 × His-tagged tail. The levels of proteins, including those after each purification step, were analyzed using sodium dodecyl sulfate-polyacrylamide gel electrophoresis (SDS-PAGE). The yield of recombinant protein was determined using the protein assay kit (500-0006) (Bio-Rad Laboratories GmbH, Vienna, Austria).

### Preparation of Water-in-Oil-in-Water Multiple Emulsion of the Oral Form of G-CSF

The water-in-oil-in-water (W/O/W) multiple emulsions were prepared using the two-step emulsified procedure with previously reported modifications (Onuki et al., 2004). Briefly, the droplets were emulsified using an equal volume of the gel solution to obtain the W/O/W emulsified particles. The composition of the three phases (inner aqueous phase:lipid phase:gel solution in the ratio 1:1:2) was as follows: inner aqueous phase, G-CSF protein solution; lipid phase, glyceryl monostearate (0.05%; v/v), span 80 (0.05%; v/v), soybean lecithin (0.05%; v/v), and soybean oil, 0.85%; v/v); and gel solution, water (1.0%; v/v), Tween 20 (0.4%; v/v), and sodium carboxymethyl cellulose (0.6%; v/v). The W/O/W-emulsified particles were stored at 4°C for further use. The formulation was prepared on ice to avoid degradation of protein at higher temperatures.

### Microarray Analysis

Microarray analysis was performed using the Agilent mouse gene expression microarray kit (Welgene Biotech, Taipei, Taiwan), following the manufacturer’s instructions. Briefly, 0.2 μg of total RNA extracted from the whole-brain lysates was labeled with Cy3 (CyDye, Agilent Technologies, Santa Clara, CA, United States) using the Low Input Quick-Amp labeling kit (Agilent Technologies, Santa Clara, CA, United States) during the *in vitro* transcription process. Cy3-labeled cRNA (0.6 μg) was fragmented to an average size of approximately 50–100 nt in fragmentation buffer at 60°C for 30 min. The fragmented and labeled cRNA was then pooled and hybridized to Agilent SurePrint Microarray (Agilent Technologies, Santa Clara, CA, United States) at 65°C for 17 h. The microarray was washed, dried using a nitrogen gun, and scanned using an Agilent microarray scanner (Agilent Technologies, Santa Clara, CA, United States) at 535 nm to detect Cy3. The scanned images were analyzed using Feature Extraction 10.7.3.1 Software (Agilent Technologies, Santa Clara, CA, United States), which is an image analysis and normalization software used to quantify signal and background intensity for each feature. The raw signal data were subjected to quantile normalization to identify differentially expressed genes. The differentially expressed genes were subjected to the enrichment test for functional assay. ClusterProfiler was used for gene ontology and Kyoto Encyclopedia of Genes and Genomes pathway analyses.

### Histological Analysis

Mice were anesthetized with Zoletil 50 (10 mg/kg i.p.; Virbac, Carros, France) at 8 h post-SCI and perfused with an intracardial infusion of phosphate-buffered saline (PBS; pH 7.4), followed by perfusion with 4% paraformaldehyde in PBS (pH 7.4) at 4°C. The mouse olfactory bulb was immediately excised, postfixed in the same fixative, and transferred to 30% sucrose in PBS until it sank. The fixed tissues were embedded at −25°C and sectioned into 20-μm coronal sections encompassing the entire olfactory bulb.

### Immunofluorescence Staining

**FIGURE 2:**
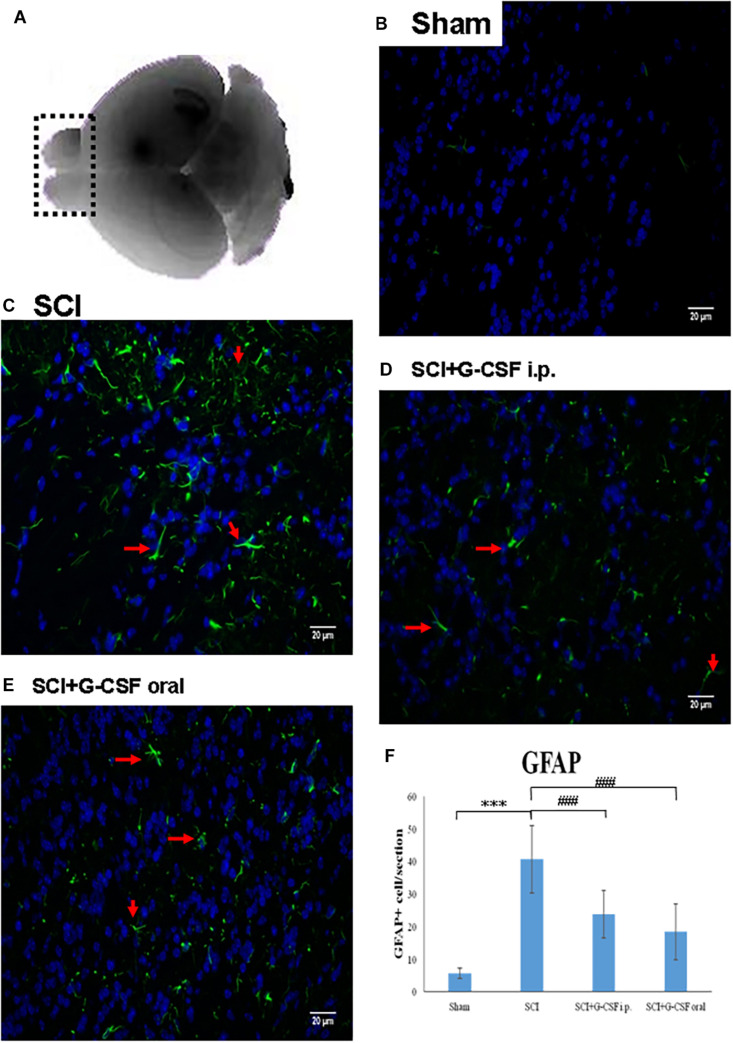
Spinal cord injury (SCI) can activate the astrocytes in the olfactory bulb at 8 h post-spinal cord hemisection injury in mice. Representative images of GFAP-stained sections of the olfactory bulbs of the sham control **(B)**, SCI **(C)**, SCI + granulocyte colony-stimulating factor (G-CSF) i.p., and SCI + G-CSF oral groups. **(A)** Schematic illustration of the olfactory bulb (marked with dashed box) of all groups subjected to mRNA, protein, and immunofluorescence analyses. The SCI group exhibited a higher number of GFAP-positive cells [as indicated by arrow in panel **(C)**] than the sham control group **(B)**. This indicated astrocytic activation and potential astrocyte-mediated inflammatory responses in the olfactory bulb at 8 h post-SCI. The immunofluorescence intensity of GFAP significantly decreased in the SCI + G-CSF i.p. [as indicated by arrow in panel **(D)**] and SCI + G-CSF oral groups [as indicated by arrow in panel **(E)**] [**(B–C)** magnification 400×]. **(E)** Vertical bars indicate the mean ± standard error of mean) number of GFAP-stained cells in each group (*n* = 3). ***P < 0.001 and ^###^*P* < 0.001.

The tissue sections of the olfactory bulb were washed thrice with PBS for 5 min and blocked with PBS containing 10% goat serum and 0.5% Triton X-100 for 1 h. Next, the sections were incubated overnight at 4°C with mouse anti-glial fibrillary acidic protein (GFAP) (MA5-12023) (1:200; Invitrogen, Carlsbad, CA, United States) in PBS containing 5% goat serum and 0.5% Triton X-100. The sections were then washed thrice with PBS containing 0.5% Triton X-100 for 5 min and incubated with goat anti-mouse IgG (H + L) cross-adsorbed secondary antibody (A11001) (1:1000) (Invitrogen, Carlsbad, CA, United States) for 3 h. After washing thrice with PBS containing 0.5% Triton X-100 for 5 min, the sections were incubated with 4′,6-diamidino-2-phenylindole (1 μg/ml; Sigma-Aldrich, St. Louis, MO, United States) for 5 min. The sections were washed, mounted on coverslips, and observed under a Zeiss AX10-Imager A1 microscope (Carl Zeiss, Thornwood, NY, United States). All images were captured using AxioVision 4.7 microscopy software (Carl Zeiss, Thornwood, NY, United States). The morphological and quantitative analyses of the olfactory bulb were performed (indicated as the region of interest within the dashed box in Figure 2A). Cell counting was performed at a magnification of 400 × on every sixth section stained using the antibodies mentioned above. Only cells exhibiting visible indications of staining were counted. All data are presented as mean ± standard error of mean of three consecutive measurements.

### Quantitative Real-Rime Polymerase Chain Reaction

Total RNA was extracted from the olfactory bulb tissues using TRIzol reagent (Invitrogen, Carlsbad, CA, United States). The RNA samples were subjected to reverse transcription using oligo-dT and SuperScript II reverse transcriptase (Invitrogen, Carlsbad, CA, United States). The quantitative real-rime polymerase chain reaction (qRT-PCR) analysis was performed using the ABI StepOne sequence detector system (Applied Biosystems, Foster City, CA, United States) with SYBR Green. The expression levels of the target genes were normalized to those of a housekeeping gene (β-actin). The primer sets and product size of each cDNA of interest were as follows: mouse IL-1β (Gene ID: 16176; accession number: NM_008361.4), 5′-AGG CTC CGA GAT GAA CAA-3′ and 5′-AAG GCA TTA GAA ACA GTC C-3′ (product size, 464 bp); IL-6 (Gene ID: 16193; accession number: NM_001314054.1), 5′-CCA CCA AGA ACG ATA GTC AA-3′ and 5′-TTT CCA CGA TTT CCC AGA-3′ (product size, 227 bp); GFAP (Gene ID: 14580; accession number: NM_001131020.1), 5′-CCA ACC CGT TCC ATA-3′ and 5′-TCC GCC TGG TAG ACA TCA-3′ (product size, 405 bp); *Olfr1494* (Gene ID: 258992; accession number: NM_146990.1), 5′-TAT GTA GTG GGC ATC CTG-3′ and 5′-GAT TGA GTA ATG GCG TGA-3′ (product size, 262 bp); *Olfr1324* (Gene ID: 258289; accession number: NM_146292.1), 5′-GCC ATC TGT CAC CCA TTA-3′ and 5′-CAA GCA AGC CTT AAC ACG-3′ (product size, 194 bp); *Olfr1241* (Gene ID: 258447; accession number: NM_146455.1), 5′-TCC ACT GCT ATC TCA CCC AA-3′ and 5′-AGG AAG CAA ACC CGC CTA-3′ (product size, 209 bp); *Olfr979* (Gene ID: 259112; accession number: NM_147108.2), 5′-GCA CCG AGT GTT TCC TGT-3′ and 5′-GAC CAT CTC ATT GGC TGA-3′ (product size, 269 bp); BDNF (Gene ID: 12064; accession number: NM_001048139.1), 5′-GGG TCA CAG CGG CAG ATA AA-3′ and 5′-GCC TTT GGA TAC CGG GAC TT-3′ (product size, 86 bp); glial cell-derived neurotrophic factor (GDNF) (Gene ID: 14573; accession number: NM_001301332.1), 5′-GGA CGC TTG GTG GTT GAT-3′ and 5′-ATG AGA ATG CTG CCG AAA-3′ (product size, 161 bp); nerve growth factor (NGF) (Gene ID: 18049; accession number: NM_001112698.2), 5′-AAG CCC ACT GGA CTA AAC T-3′ and 5′-GTC TTA TCT CCA ACC CAC A-3′ (product size, 340 bp); Nestin (Gene ID: 18008; accession number: NM_016701.3), 5′-CCC TGA AGT CGA GGA GCT G-3′ and 5′-CTG CTG CAC CTC TAA GCG A-3′ (product size 166 bp); and β-actin (Gene ID: 11461; accession number: NM_007393.5), 5′-CTG TCC CTG TAT GCC TCT G-3′ and 5′-ATG TCA CGC ACG ATT TCC-3′ (product size, 218 bp).

### Immunoblotting

Total protein was extracted from the mouse olfactory bulb tissues in a lysis buffer containing 0.8% NaCl, 10% glycerol, 0.1% SDS, 1% Triton X-100, 20 mM Tris–HCl, and 1 mM phenylmethylsulfonyl fluoride. The lysates were centrifuged at 13,000 *g* and 4°C for 10 min. The total protein content in the supernatant was determined using a protein assay kit (500-0006) (Bio-Rad Laboratories GmbH, Vienna, VA, United States). The sample was then boiled for 5 min, and 20 μl (containing 20 μg of protein) aliquot of the sample was subjected to SDS-PAGE using a 12% gel. The resolved proteins were electroblotted to a nitrocellulose membrane. The membrane was blocked with the blocking reagent and incubated with the following primary antibodies for 12 h at 4°C: mouse anti-GFAP (MA5-12023) (1:2500) (Invitrogen, Carlsbad, CA, United States); rabbit anti-Nestin (tcna6785) (1:2500) (Taiclone, Taipei, Taiwan); rabbit anti-IL-6 (tcba214) (1:1000) (Taiclone, Taipei, Taiwan); rabbit anti-IL-1β (tcea9325) (1:1000) (Taiclone, Taipei, Taiwan); rabbit anti-β-actin (ab8227) (1:1000) (Abcam, Cambridge, MA, United States). Next, the membrane was washed thrice with PBS containing 0.05% Tween-20 for 5 min and incubated with goat anti-rabbit IgG (H + L) horseradish peroxidase (HRP)-conjugated secondary antibody (31460) (1:5000) (Invitrogen, Carlsbad, CA, United States), and goat anti-mouse IgG (H + L) HRP-conjugated secondary antibody (31430) (1:5000) (Invitrogen, Carlsbad, CA, United States) for 2 h. Immunoreactive signals were detected using SuperSignal^TM^ West Pico Chemiluminescent Substrate (Catalog: 34080, Thermo Scientific^TM^, Waltham, MA, United States). The protein bands were visualized and quantified using ImageQuant^TM^ LAS 4000 (GE Healthcare Life Sciences, Marlborough, MA, United States).

### Statistical Analyses

An independent two-sample *t*-test was used to compare the means of the two groups for microarray analysis. To assess differential mRNA and protein expression, three or more independent groups were compared using one-way analysis of variance. In cases where the differences were apparent, multiple comparisons were made using the Newman–Keuls method. Data are presented as mean ± standard error of mean. All statistical analyses were two-sided tests with the level of significance set at 0.05. All statistical analyses were performed using GraphPad Prism software version 5.0 (GraphPad Software, Inc., La Jolla, CA, United States).

## Results

### SCI Downregulated the mRNA Levels of Olfactory Receptors in the Brain

Olfactory dysfunction can be an early indicator of neurological disorders, such as SCD or neurodegenerative diseases. SCI can lead to SCD or neurodegenerative diseases. This study examined if impaired olfaction is a risk factor for SCI-mediated neurodegeneration in the brain. An animal model of SCI was established to examine the effect of SCI on olfactory function. The whole-brain lysates of the mice from the sham control, SCI, and SCI + G-CSF i.p. groups (*n* = 3 for each group) were subjected to microarray analysis at 8 h post-SCI.

The microarray analysis revealed that the mRNA expression levels of *Olfr1494*, *Olfr979*, *Olfr424*, *Olfr122*, *Olfr1395*, *Olfr689*, *Olfr1457*, *Olfr384*, *Olfr969*, *Olfr945*, *Olfr788*, and *Olfr1339* (olfactory receptors) in the brain of the SCI group were significantly downregulated when compared to those in the brain of the control group at 8 h post-SCI (^∗^*P* < 0.05 for all) (Supplementary Table 1).

As shown in Table 1, the mRNA expression levels of *Olfr1494*, *Olfr979* (^∗^*P* < 0.05 and ^∗^*P* < 0.05, respectively), *Mc3r* (G-protein-coupled receptor) (^∗^*P* < 0.05), *Ppp3cb* (regulator of calcium ion-regulated exocytosis), *Drd4* (dopamine neurotransmitter receptor), and *Gabrr3* (GABA-A receptor) in the brain of the SCI group were markedly lower than those in the brain of the sham control group. The mRNA expression levels of *Olfr1494* (^∗^*P* < 0.05), *Olfr979* (^∗^*P* < 0.05), *Mc3r*, *Ppp3cb*, *Drd4*, and *Gabrr3* in the SCI + G-CSF i.p. group were higher than those in the SCI group.

**TABLE 1:** Results of mRNA microarray analysis of mouse whole brain 8 h following SCIs.

Gene	SCI/sham (fold change)	Significance	SCI + G-CSF/SCI (fold change)	Significance	Function
*Olfr1494*	0.323	*	3.607	*	Olfactory reception
*Olfr979*	0.262	*	3.931	*	Olfactory reception
*Mc3r*	0.176	*	2.401		G-protein coupled receptor
*Mc5r*	2.142		0.460		G-protein coupled receptor
*Ppp3cb*	0.459		2.267		Calcium ion regulated exocytosis
*Drd4*	0.372		2.636		Dopamine neurotransmitter receptor
*Gabrr3*	0.400		2.378		GABA-A receptor
*Ppp2r3a*	5.757	*	0.179	*	Protein binding, bridging

*P < 0.05.

Compared with those in the brain of the control group mice, the expression levels of *Mc5r* (G-protein-coupled receptor) and *Ppp2r3a* (regulator of protein binding and bridging) (^∗^*P* < 0.05) were upregulated in the brain of the SCI group mice at 8 h post-SCI. Additionally, treatment with G-CSF downregulated the expression of *Mc5r* and *Ppp2r3a* (^∗^*P* < 0.05) in SCI mice.

These findings indicated that acute SCIs elicit inflammatory responses in the spinal cord and the brain. Additionally, SCI can damage the olfactory bulb and consequently decrease the number of olfactory receptors.

### SCI Promoted Inflammation in the Brain Through the Activation of Astrocytes

The results of microarray analysis revealed that SCI promoted brain damage as early as 8 h post-SCI, which was characterized by downregulated expression of olfactory receptors. Olfactory defects are highly correlated with neurodegenerative diseases. Hence, this study focused on the pathology of the olfactory bulb in SCI animals (shown in the dashed box in Figure 2A). The ability of acute SCIs to induce inflammatory responses in the olfactory bulb and the subsequent neurodegeneration in the brain was examined.

The mice were divided into the following four groups: sham control, SCI, SCI + G-CSF i.p., and SCI + G-CSF oral (*n* = 15 for each group). Astrocytes, which are the resident innate immune cells in the central nervous system (CNS), can be reactivated to mediate neuroinflammation. The reactivation is characterized by astrocytic hypertrophy and the release of various pro-inflammatory mediators. The GFAP immunofluorescence (arrow in Figure 2C) in the olfactory bulb was higher and denser in the SCI group (^∗∗∗^*P* < 0.001; Figure 2F) than in the sham control group (Figure 2B). This indicated that astrocytes are reactivated in the olfactory bulb at an early phase post-SCI, although the lesion was located at the spinal cord.

The GFAP fluorescence signal in the SCI group (###*P* < 0.001 and ###*P* < 0.001, respectively; Figure 2F) was higher and denser than that in the SCI + G-CSF i.p. (Figure 2D) and SCI + G-CSF oral groups (Figure 2E).

### SCI Promoted Neuroinflammation in the Olfactory Bulb *in vivo*

**FIGURE 3:**
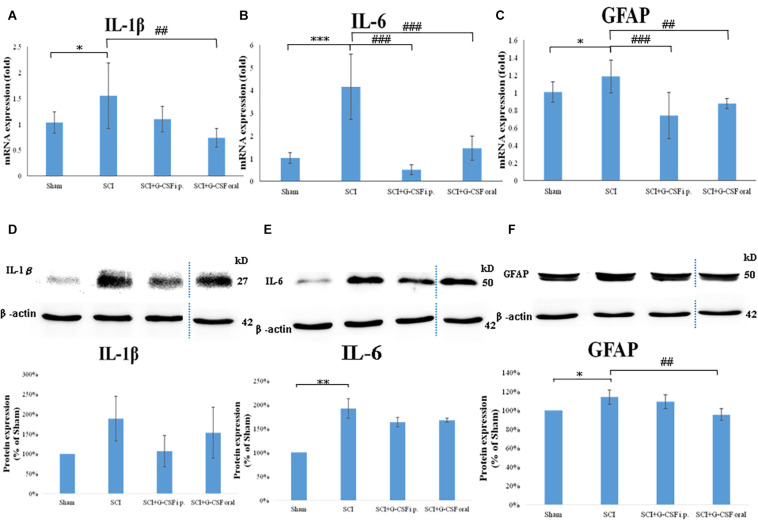
Neuroinflammation in the mouse olfactory bulb at 8 h post-spinal cord injury (SCI). **(A–C)** The mRNA expression levels of IL-1β **(A)**, IL-6 **(B)**, and GFAP **(C)** in the olfactory bulb of the sham control, SCI, SCI + granulocyte colony-stimulating factor (G-CSF) i.p., and SCI + G-CSF oral groups at 8 h post-SCI. Vertical bars indicate the mean ± standard error of the mean (SEM) (*n* = 6 for each group). ^∗^*P* < 0.05, ^∗∗∗^*P* < 0.001, ##*P* < 0.01, and ###*P* < 0.001. **(D–F)** The protein expression levels of IL-1β **(D)**, IL-6 **(E)**, and GFAP **(F)** in the olfactory bulb of the four experimental groups at 8 h post-SCI. Representative immunoblots of IL-1β, IL-6, GFAP, and β-actin (internal control) are shown in the upper panel. The lower panel indicates the ratio of target protein band intensity to β-actin protein band intensity relative to the control group (mean ± SEM). G-CSF mitigates SCI-induced neuroinflammation in the olfactory bulb as evidenced by the decreased expression of IL-1β, IL-6, and GFAP. Vertical bars indicate mean ± SEM (*n* = 6 for each group). ^∗^*P* < 0.05, ^∗∗^*P* < 0.01, and ##*P* < 0.01.

To analyze the effect of SCI on neuroinflammation, the olfactory bulb of the mice in the four groups (*n* = 6 for each group) was subjected to qRT-PCR analysis. Each experiment was performed three times. SCI promotes the activation of astrocytes and the release of pro-inflammatory cytokines. The mRNA expression levels of IL-1β (^∗^*P* < 0.05; Figure 3A), IL-6 (^∗∗∗^*P* < 0.001; Figure 3B), and GFAP (^∗^*P* < 0.05; Figure 3C) in the SCI group were upregulated when compared with those in the sham control group.

Treatment with G-CSF alleviated neuroinflammation in the olfactory bulb in mice with spinal cord hemisection. The mRNA expression levels of IL-1β, IL-6 (###*P* < 0.001; Figure 3B), and GFAP (###*P* < 0.001; Figure 3C) were significantly lower in the SCI + G-CSF i.p. group than in the SCI group. Similarly, the mRNA levels of IL-1β (##*P* < 0.01; Figure 3A), IL-6 (###*P* < 0.001; Figure 3B), and GFAP (##*P* < 0.01; Figure 3C) in the olfactory bulb were lower in the SCI + G-CSF oral group than in the SCI group.

Western blot analysis was performed to verify neuroinflammation within the olfactory bulb from four groups of mice (*n* = 6 for each group) at the protein level. Unlike in the sham control group, enhanced astrocyte reactivation (elevated GFAP; ^∗^*P* < 0.05; Figure 3F) and glial activation-induced pro-inflammatory cytokine production (upregulated expression levels of IL-1β; Figure 3D and IL-6; ^∗∗^*P* < 0.001; Figure 3E) at 8 h post-SCI was observed in the SCI group. Additionally, the SCI + G-CSF i.p. group exhibited downregulated levels of IL-1β (Figure 3D), IL-6 (Figure 3E), and GFAP (Figure 3F). Similarly, the SCI + G-CSF oral group exhibited downregulated levels of IL-1β (Figure 3D), IL-6 (Figure 3E), and GFAP (## *P* < 0.001; Figure 3F).

Previous studies have reported that neurodegeneration in the brain is correlated with olfactory deficits and SCI. The findings of this study indicated that SCI can lead to early neuroinflammation of the olfactory bulb.

### G-CSF Mitigates SCI-Induced Downregulated mRNA Expression of Olfactory Receptors

**FIGURE 4:**
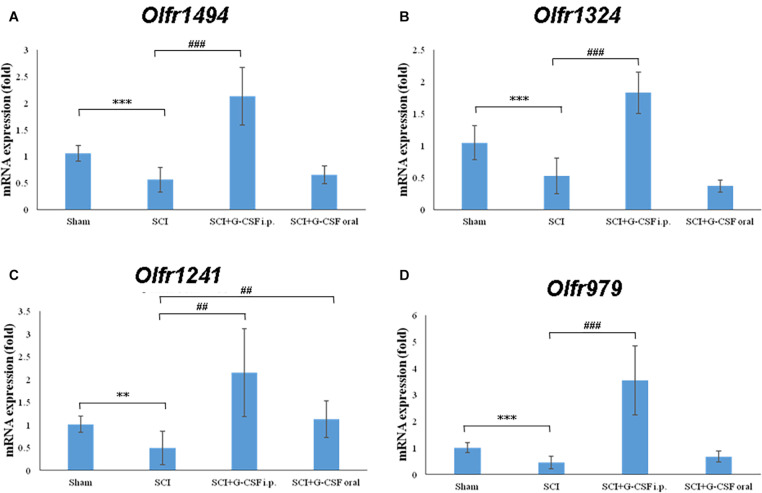
Spinal cord injury (SCI) decreases the expression of olfactory receptors in the olfactory bulb at 8 h post-SCI. The mRNA expression levels of **(A)** *Olfr1494*, **(B)** *Olfr1324*, **(C)** *Olfr1241*, and **(D)** *Olfr979* in the olfactory bulb of the sham control, SCI, SCI + G-CSF i.p., and SCI + G-CSF oral groups. Vertical bars indicate mean ± standard error of mean (*n* = 6 in each group). ^∗∗^*P* < 0.01, ^∗∗∗^*P* < 0.001, ##*P* < 0.01, and ###*P* < 0.001.

The qRT-PCR analysis revealed that the expression levels of the olfactory receptors *Olfr1494* (^∗∗∗^*P* < 0.001; Figure 4A), *Olfr1324* (^∗∗∗^*P* < 0.001; Figure 4B), *Olfr1241* (^∗∗^*P* < 0.01; Figure 4C), and *Olfr979* (^∗∗∗^*P* < 0.001; Figure 4D) in the SCI group were significantly downregulated at 8 h post-SCI when compared with those in the sham control group.

The mRNA expression levels of *Olfr1494* (###*P* < 0.001; Figure 4A), *Olfr1324* (###*P* < 0.001; Figure 4A), *Olfr1241* (##*P* < 0.01; Figure 4C), and *Olfr979* (###*P* < 0.001; Figure 4D) in the olfactory bulb of the SCI + G-CSF i.p. group were significantly upregulated when compared with those in the olfactory bulb of the SCI group. Compared with that in the sham control and SCI groups, the mRNA expression level of *Olfr1241* in the olfactory bulb was significantly upregulated in the SCI + G-CSF oral group (## *P* < 0.01; Figure 4C).

These results indicate that in the ultrarapid stage of CNS injury (including SCI), the mRNA expression of olfactory receptors is downregulated, which may further contribute to olfactory dysfunction and potential degeneration of the olfactory neural network and consequently neurodegeneration. Previous studies have reported that the olfactory bulb is a sentinel station for neurodegeneration in the brain. G-CSF can mitigate SCI-induced changes in mRNA expression and achieve early preventive effects.

### G-CSF Mitigates SCI-Induced Downregulation of mRNA and Protein Levels of Nestin and Neurotrophic Factors in the Olfactory Bulb

**FIGURE 5:**
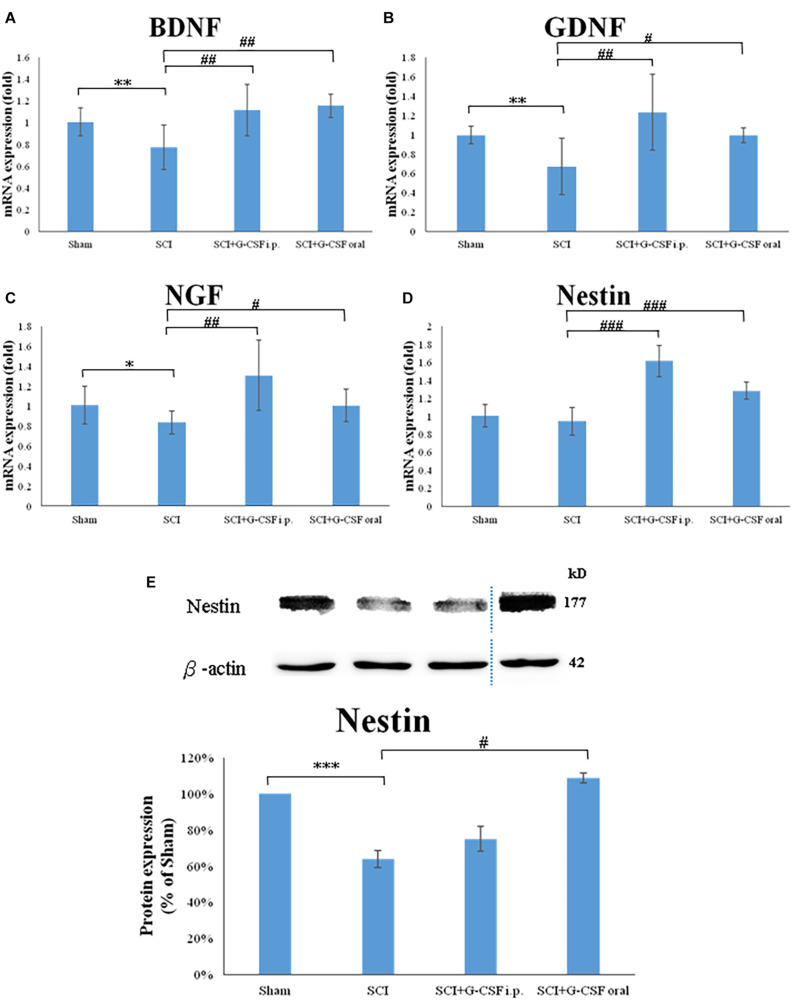
Spinal cord injury (SCI) downregulates the expression of neurotrophic factors and the number of neural stem cells (NSCs) in the olfactory bulb at 8 h post-SCI. **(A–D)** The mRNA expression levels of BDNF **(A)**, GDNF **(B)**, NGF **(C)**, and Nestin (NSC marker) **(D)** in the olfactory bulb of the sham control, SCI, SCI + granulocyte colony-stimulating factor (G-CSF) i.p., and SCI + G-CSF oral groups. **(E)** The protein expression levels of Nestin in the olfactory bulb of the four experimental groups at 8 h post-SCI. The upper panel shows the immunoblot of Nestin (177 kDa). β-Actin (42 kDa) served as an internal control. The lower panel indicates the ratio of Nestin protein band intensity to β-actin protein band intensity relative to the control group. Vertical bars indicate mean ± standard error of mean for mRNA **(A–D)** or protein expression **(E)** [*n* = 6 in each group for panels **(A–E)**]. ^∗^*P* < 0.05, ^∗∗^*P* < 0.01, ^∗∗∗^*P* < 0.001, #*P* < 0.05, ##*P* < 0.01, and ###*P* < 0.001.

The SCI group exhibited significantly downregulated mRNA expression levels of BDNF (^∗∗^*P* < 0.01; Figure 5A), GDNF (^∗∗^*P* < 0.01; Figure 5B), NGF (^∗^*P* < 0.05; Figure 5C), and Nestin (marker of NSC) (Figure 5D) when compared with the sham control group at 8 h post-SCI.

The mRNA expression levels of BDNF (##*P* < 0.01; Figure 5A), GDNF (##*P* < 0.01; Figure 5B), NGF (##*P* < 0.01; Figure 5C), and Nestin (###*P* < 0.001; Figure 5D) in the olfactory bulb of the SCI + G-CSF i.p. group were upregulated when compared with those in the olfactory bulb of the SCI group at 8 h post-SCI. Compared with those in the olfactory bulb of the SCI group, the mRNA expression levels of BDNF (##*P* < 0.01; Figure 5A), GDNF (#*P* < 0.05; Figure 5B), NGF (#*P* < 0.05; Figure 5C), and Nestin (###*P* < 0.001; Figure 5D) were significant upregulated in the olfactory bulb of the SCI + G-CSF oral group.

The protein expression of Nestin was quantified using western blotting. Compared with that in sham control, the expression of Nestin was significantly downregulated in the SCI group (^∗∗∗^*P* < 0.001; Figure 5E). However, the expression levels of Nestin in the SCI + G-CSF i.p. and SCI + oral groups (#*P* < 0.05; Figure 5E) were higher than those in the SCI group.

These findings indicated that neuroinflammation of the olfactory bulb partly affects the neurogenesis-associated processes, including the generation of NSCs and the production of associated neurotrophic factors. G-CSF treatment may promote the generation of NSCs and the production of neurotrophic factors and consequently mitigate SCI-induced downregulated olfactory receptors and olfactory dysfunction. Thus, G-CSF may mitigate neurodegeneration in other areas of the brain.

## Discussion

Olfactory dysfunction is an indicator of various neurological diseases, such as PD, AD, and depression. Patients with neurodegeneration exhibit significantly impaired odor sensitivity, discrimination, and identification (Altinayar et al., 2014; Negoias et al., 2016; Yoo et al., 2018). Neurological diseases, including PD, AD, and depression, are associated with dysfunctional serotonergic and dopaminergic neurotransmission (Croy and Hummel, 2017). The major transmission routes of the serotonin and dopamine systems include the orbitofrontal cortex, hippocampus, striatum, and amygdala (Drevets, 2007; Hamilton et al., 2008; Gabbay et al., 2013). Additionally, odor molecules are initially sensed by the olfactory receptors in the olfactory bulb. Next, the olfactory signal passes through the prefrontal cortex, hippocampus, striatum, and amygdala (Yang et al., 2011; Negoias et al., 2016; Croy and Hummel, 2017). The transmission route of the olfactory signals overlaps with the dopamine and serotonin pathways. The structural and functional impairments among these brain regions lead to dysfunctional neurotransmission and consequently result in impaired olfactory conduction. This explains the correlation between olfactory dysfunction and neurodegeneration.

Several animal and human experiments have confirmed that the olfactory bulb, which regulates the olfactory receptors, plays an important role in neurodegeneration in the brain. The volume of the olfactory bulb is correlated with depression severity (Negoias et al., 2010). Patients with depression who do not exhibit improvements with psychotherapy are associated with severe olfactory bulb atrophy (Croy and Hummel, 2017).

Various studies have reported that pathogens, including viruses, bacteria, prions, and toxins, can enter the brain directly through the olfactory pathway and damage the neural structure (Doty, 2008). The olfactory epithelium, which is in direct contact with the external environment, is separated from the external environment only by a thin layer of mucus. Thus, the olfactory system is susceptible to various external factors. The olfactory bulb has been considered as the entry point for pathogens, which spread to the brain through the olfactory pathway and consequently cause pathological changes (Rey et al., 2018). Viruses, which are a risk factor for neurodegenerative diseases, can penetrate olfactory receptor neurons. Next, viruses are transported through axons in the space around the nerves, spread through the cribriform plate, and transported to the subarachnoid space and remote brain areas (Dando et al., 2014). Moreover, pathological protein aggregation in the olfactory bulb is detected earlier than that in other regions (Rey et al., 2018). This indicates that the olfactory bulb is vulnerable to environmental insults and that it is involved in early neurodegenerative diseases.

In addition to regulating olfactory function, the olfactory bulb is one of the sites at which NSCs are stored (Pagano et al., 2000). The hippocampus is the other site at which NSCs are stored (Rolando and Taylor, 2014). The characteristics of NSCs include self-renewal and multipotent differentiation into neurons or glial cells. NSCs have been isolated from the olfactory bulb, cortex, hippocampus, or subventricular zone (SVZ) of lateral ventricles of the brain (Marei et al., 2015). Generally, neural progenitors formed during neurogenesis in the SVZ migrate forward along the rostral migratory stream (RMS) (Whitman et al., 2009), and combine neural circuits to develop into interneurons in the olfactory bulb (kay-Sim, 2010). NSCs differentiate into neural cells, including neurons and glial cells, in response to environmental stimuli (Alizadeh et al., 2017). Additionally, NSCs can regenerate olfactory receptor neurons (Godoy et al., 2015). Chronic inflammation may impair the neurogenesis of olfactory NSCs (Rustenhoven and Kipnis, 2019).

Complex mechanisms are involved in neuroblast migration in the RMS. Astrocytes, astrocyte-released growth factors, and neurotrophic factors are the regulatory factors that determine neuroblast migration. The blood vessels are reported to support forward motility during neuroblast migration (Motamed et al., 2019). The generation of blood vessels is mediated by astrocyte-secreted vascular endothelial growth factor (Bozoyan et al., 2012). Astrocytes located at the boundary of RMS undergo hypertrophy and branching and promote the wrapping of neuroblasts along with blood vessels to form glial tubes, which results in the separation of the neuroblasts from the surrounding tissues (Snapyan et al., 2009; Motamed et al., 2019). BDNF, which is secreted from the vasculature, increases the number and motility of neuroblasts (Chiaramello et al., 2007). Astrocytes regulate the activity and migration of neuroblasts through BDNF (Snapyan et al., 2009). GDNF serves as a directional cue for the chemotaxis of neuroblasts from SVZ to the olfactory bulb (Paratcha et al., 2006).

Neural stem cells migrate toward lesioned sites upon CNS injury and promote neurogenesis (Imitola et al., 2004; Bai et al., 2018). Similar to the migration mechanism in RMS, neuroblasts use blood vessels as a physical guide to migrate from SVZ to the lesion site (Kaneko et al., 2017). Newly generated neuroblasts are recruited into blood vessels and redirected to the lesioned site *via* the chemoattractive/trophic factors, such as BDNF, stromal-cell-derived factor-1α (SDF-1α), and metalloproteinase-9 (MMP-9) (Ghashghaei et al., 2007; Grade et al., 2013). However, the intrinsic neuronal repair mechanism is not effective. For example, the endogenous BDNF levels are low and consequently the regeneration process is impaired.

A rat model of systemic lipopolysaccharide (LPS) treatment exhibited inflammatory response and upregulation of cytokines in the olfactory bulb at 6 h post-LPS administration (Doursout et al., 2013). The findings of this study indicated that the olfactory bulb is vulnerable to environmental insults, such as damage from the remote periphery or CNS. This study elucidated the manifestations of neuroinflammatory responses in the olfactory bulb and the potential mechanisms leading to olfactory dysfunction, including the downregulation of olfactory receptors, the production of NSC, and the secretion of neurotrophic factors at 8 h post-SCI. In this study, SCI promoted neuroinflammation in the olfactory bulb, which was characterized by the activation of resident astrocytes and the subsequent release of pro-inflammatory cytokines. Astrocyte reactivation in the olfactory bulb may promote glial stimulation and whole-brain inflammation and impair NSC regeneration. Aberrant activation of innate immune cells and the inflamed brain may lead to neurodegenerative changes in the brain (Kung and Lin, 2021a, b). The decreased number of olfactory receptors may reduce the turnover rate toward the olfactory bulb, which leads to atrophy of the olfactory bulb. The decrease in the olfactory bulb volume eventually leads to a decline in the signal from the olfactory bulb to the amygdala, hippocampus, striatum, and orbitofrontal cortex, which exacerbates neurodegeneration (Croy and Hummel, 2017). Moreover, the olfactory bulb in the SCI group exhibited decreased Nestin expression and downregulated production of neurotrophic factors, including BDNF, NGF, and GDNF. The key factors that promote neurogenesis, including NSCs, supportive neurotrophic/chemotactic factors, and protective astrocytes for constructing glial tubes, are dysregulated, which promotes neurodegeneration in the brain. We hypothesized that the ultrarapid stage after CNS injury [even lesions located at sites distant from the brain (such as SCI)] initiates degenerative changes in the brain, which are characterized by pathological changes in the olfactory bulb, including astrocyte-driven neuroinflammation, olfactory dysfunction, and impaired production of NSCs and neurotrophic factors. G-CSF can mitigate these pathological changes in the olfactory bulb.

The olfactory bulb and the related tracts and projections to specific brain regions, including the hippocampus and amygdala, are responsible for the storage and recovery of memory and emotional regulation (Roberts et al., 2016). Neurodegenerative insults can damage the nuclei that produce acetylcholine, dopamine, and norepinephrine and consequently decrease the production of choline acetyltransferase, which leads to cholinergic, dopaminergic, and noradrenergic deficiencies (Mesholam et al., 1998; Doty, 2012). Thus, olfactory dysfunction is exacerbated with subsequent cognitive decline and dementia. In the amyloid beta-induced AD rat model, G-CSF downregulated the expression of acetylcholinesterase in the brain (Prakash et al., 2013b). G-CSF inhibits the acetylcholinesterase-mediated hydrolysis of acetylcholine into choline and acetic acid, which results in enhanced plasma concentration of acetylcholine. In the MPTP-induced PD mouse model, G-CSF inhibited MPTP-induced cell death of dopaminergic substantia nigra neurons and attenuated the reduction of striatal dopamine (Meuer et al., 2006). G-CSF decreases the reuptake of norepinephrine in the peripheral sympathetic neurons and consequently increases the release of norepinephrine (Lucas et al., 2012). In this study, G-CSF increased the number of NSCs as evidenced by increased expression of Nestin and neurotrophic factors, including BDNF, GDNF, and NGF, in the olfactory bulb. The administration of recombinant BDNF in the brain promotes neurogenesis in the striatum and olfactory bulb (Zigova et al., 1998; Benraiss et al., 2001) in addition to enhancing neuroblast migration to the lesion site in the mouse injury model (Grade et al., 2013). The beneficial effects of epidermal growth factor and fibroblast growth factor-2 on neurogenesis in SVZ and the olfactory bulb have been demonstrated in the PD animal model (Winner et al., 2008). Olfactory deficiency, which is an early marker of neurodegeneration in the brain, is associated with various functional nuclei of olfactory projection that are involved in cognition, memory, and dysfunction of neurotransmission. Therefore, G-CSF can prevent olfactory dysfunction, initial inflammation of the olfactory bulb and brain, and the subsequent neurodegeneration in the brain.

This study is associated with several limitations. In this study, the pathological changes of the olfactory bulb were investigated in the ultrarapid phase after CNS injury to verify the role of the olfactory bulb as an early lesion site of neurodegeneration in the brain. This study demonstrated the local benefits of G-CSF in the early stage of inflammation in the olfactory bulb. Although previous studies have reported the correlation between the olfactory bulb and associated functional nucleus, further studies are needed to examine the effect of the inflammation in the olfactory bulb on the entire brain and the consequent development of neurodegenerative diseases. Additionally, this study examined the markers of NSCs (Nestin) and the production of BDNF. However, neurogenesis and migration of NSCs in the olfactory bulb or SVZ have not been demonstrated. The pathological manifestations of the olfactory bulb were demonstrated in an early stage of CNS injury. Although the mRNA levels varied, the corresponding protein levels did not exhibit a marked change. However, the olfactory bulb exhibited inflammation and impaired neurogenesis at 8 h post-SCI. The novel findings of this study enable the development of clinical interventions for neurodegenerative diseases.

## Conclusion

Previous studies have reported that the olfactory bulb and olfactory dysfunction are involved in neurodegenerative diseases. This study demonstrated that CNS injury (even SCI located at a distant site from the brain) promotes inflammatory response in the olfactory bulb at an early stage, which is accompanied by downregulation of olfactory receptors, impaired neurogenesis, and decreased production of NSCs and BDNF. G-CSF administration can mitigate the pathological changes in the olfactory bulb at an early stage of CNS injury in mice with spinal cord hemisection. The findings of this study will contribute to further studies on the pathophysiological mechanisms of early neurodegenerative diseases involving the olfactory bulb and aid in the development of early clinical drug interventions.

## Data Availability Statement

The raw data supporting the conclusions of this article will be made available by the authors, without undue reservation.

## Ethics Statement

The animal study was reviewed and approved by Animal Care and Ethics Committee of National Ilan University, Yilan, Taiwan.

## Author Contributions

M-SL: writing-original draft, writing-review and editing, and formal analysis. I-HC: project administration and methodology. C-CL: resources, funding acquisition, conceptualization, and formal analysis. All authors contributed to the article and approved the submitted version.

## Conflict of Interest

The authors declare that the research was conducted in the absence of any commercial or financial relationships that could be construed as a potential conflict of interest.
