# Supplementary material for: Ultrarapid Inflammation of the Olfactory Bulb After Spinal Cord Injury: Protective Effects of the Granulocyte Colony-Stimulating Factor on Early Neurodegeneration in the Brain
Source: Front Aging Neurosci. 2021 Jun 25;13:701702. doi: 10.3389/fnagi.2021.701702 (PMC8267925; doi:10.3389/fnagi.2021.701702)
Supplement: Supplementary file 1 [file Data_Sheet_1.DOC]

Results of mRNA microarray analysis of mouse whole brain 8 hs following SCIs

| Gene | SCI/sham  (Fold Change) | Significance | SCI+G-CSF/SCI  (Fold Change) | Significance | Function |
| --- | --- | --- | --- | --- | --- |
| *Olfr1494* | 0.323 | * | 3.607 | * | Olfactory reception |
| *Olfr979* | 0.262 | * | 3.931 | * | Olfactory reception |
| *Olfr424* | 0.201 | * |  |  | Olfactory reception |
| *Olfr122* | 0.217 | * |  |  | Olfactory reception |
| *Olfr1395* | 0.240 | * |  |  | Olfactory reception |
| *Olfr689* | 0.243 | * |  |  | Olfactory reception |
| *Olfr1457* | 0.263 | * |  |  | Olfactory reception |
| *Olfr384* | 0.268 | * |  |  | Olfactory reception |
| *Olfr969* | 0.284 | * |  |  | Olfactory reception |
| *Olfr945* | 0.327 | * |  |  | Olfactory reception |
| *Olfr788* | 0.332 | * |  |  | Olfactory reception |
| *Olfr1339* | 0.332 | * |  |  | Olfactory reception |
| *Olfr399* |  |  | 3.157 | * | Olfactory reception |
| *Olfr693* |  |  | 3.165 | * | Olfactory reception |
| *Olfr39* | 5.143 | * | 0.246 | * | Olfactory reception |
| *Olfr806* |  |  | 0.272 | * | Olfactory reception |
| *Wwp2* | 0.131 | * |  |  | WW domain containing E3 ubiquitin protein ligase 2 |
| *Cblc* | 0.301 | * |  |  | Casitas B-lineage lymphoma c |
| *Mc3r* | 0.176 | * | 2.401 |  | G-protein coupled receptor |
| *Mc5r* | 2.142 |  | 0.460 |  | G-protein coupled receptor |
| *Ppp3cb* | 0.459 |  | 2.267 |  | Calcium ion regulated exocytosis |
| *Drd4* | 0.372 |  | 2.636 |  | Dopamine neurotransmitter receptor |
| *Gabrr3* | 0.400 |  | 2.378 |  | Gamma-aminobutyric acid(GABA)-A receptor |
| *Ppp2r3a* | 5.757 | * | 0.179 | * | Protein binding, bridging |
| *Gabrg3* |  |  | 0.095 | ** | GABA- A receptor |
| *Camkk2* |  |  | 0.177 | * | Calcium/Calmodulin-dependent protein kinase kinase 2, |
| *Adora2a* | 5.746 | * |  |  | Adenosine A 2A receptor, Regulation of glutamate and dopamine release |
| *Cr2* | 4.703 | * |  |  | Complement receptor 2 |
| *Prpf19* | 4.424 | * |  |  | Pre-mRNA processing factor 19 |
| *Tph2* |  |  | 3.822 | * | Tryptophan hydroxylase 2 |
| *Slc6a4* |  |  | 3.948 | * | Sodium-dependent serotonin transporter |

*P < 0.05
